# Combining noninvasive brain stimulation with behavioral pharmacology methods to study mechanisms of substance use disorder

**DOI:** 10.3389/fnins.2023.1150109

**Published:** 2023-07-24

**Authors:** Michael J. Wesley, Joshua A. Lile

**Affiliations:** ^1^Department of Behavioral Science, College of Medicine, University of Kentucky, Lexington, KY, United States; ^2^Department of Psychiatry, College of Medicine, University of Kentucky, Lexington, KY, United States; ^3^Department of Psychology, College of Arts and Sciences, University of Kentucky, Lexington, KY, United States

**Keywords:** brain stimulation, addiction, neural technology, behavioral pharmacology, cannabis

## Abstract

Psychotropic drugs and transcranial magnetic stimulation (TMS) are effective for treating certain psychiatric conditions. Drugs and TMS have also been used as tools to explore the relationship between brain function and behavior in humans. Combining centrally acting drugs and TMS has proven useful for characterizing the neural basis of movement. This combined intervention approach also holds promise for improving our understanding of the mechanisms underlying disordered behavior associated with psychiatric conditions, including addiction, though challenges exist. For example, altered neocortical function has been implicated in substance use disorder, but the relationship between acute neuromodulation of neocortex with TMS and direct effects on addiction-related behaviors is not well established. We propose that the combination of human behavioral pharmacology methods with TMS can be leveraged to help establish these links. This perspective article describes an ongoing study that combines the administration of delta-9-tetrahydrocannabinol (THC), the main psychoactive compound in cannabis, with neuroimaging-guided TMS in individuals with problematic cannabis use. The study examines the impact of the left dorsolateral prefrontal cortex (DLPFC) stimulation on cognitive outcomes impacted by THC intoxication, including the subjective response to THC and the impairing effects of THC on behavioral performance. A framework for integrating TMS with human behavioral pharmacology methods, along with key details of the study design, are presented. We also discuss challenges, alternatives, and future directions.

## Highlights

– Combining drug administration and noninvasive brain stimulation has proven useful for characterizing the neural basis of movement.
– Characterizing the neural basis of addiction behavior is challenging and the links between neocortical function and addiction related behavior are poorly understood.
– Integrating noninvasive brain stimulation into a behavioral pharmacology framework can help establish a better understanding of the neural basis of addiction behavior.

## 1. Introduction

Psychotropic drugs and transcranial magnetic stimulation (TMS) are effective for treating psychiatric conditions, including major depressive disorder (Perera et al., 2016; Cipriani et al., 2018), obsessive–compulsive disorder (Pittenger and Bloch, 2014; Rapinesi et al., 2019), post-traumatic stress disorder (de Moraes Costa et al., 2020; Harris and Reece, 2021) and tobacco use disorder (Nagano et al., 2019; BrainsWay, 2020). Drugs acting on the central nervous system (CNS) and TMS have also been used as tools to explore the relationship between brain function and behavior. A prominent example is prior work combining TMS with centrally acting drugs to characterize the neural basis of motor behavior (Korchounov and Ziemann, 2011; Ziemann, 2013). With this pharmaco-TMS approach, the ability of specific TMS protocols to directly modulate cellular activity in the brain to produce acute changes in behavior is established (Chipchase et al., 2012). Next, pharmacologically selective drugs are administered to determine their impact on TMS-induced behavior, thereby uncovering mechanisms of motor function. This approach has provided extensive evidence of the acute interactions between TMS and CNS drugs on behavior (Ziemann, 2011; Nitsche et al., 2012) and these findings support the use of these combined interventions to improve our understanding of the neural basis of psychiatric conditions, including substance use disorder (SUD).

SUD is described as a chronic, relapsing condition characterized by continued drug use despite its negative consequences. SUDs have been linked to abnormal function in brain networks related to reward, stress, and self-control (Koob and Volkow, 2016; Uhl et al., 2019; Ceceli et al., 2022). As highlighted in several prominent review articles non-invasive brain stimulation has shown promise as an intervention for addiction (Feil and Zangen, 2010; Gorelick et al., 2014; Yavari et al., 2016; Dunlop et al., 2017; Coles et al., 2018; Hanlon et al., 2018; Ekhtiari et al., 2019; Steele, 2020a,b). As such, there is substantial interest in using TMS to probe function in brain regions thought to underly facets of SUD, but challenges exist. Unlike the relatively well understood relationship between primary motor cortex function and motor behavior, the effect of neocortical neuromodulation with TMS on addiction-related behavior is not well established (Spagnolo and Goldman, 2017). The combination of TMS with abused drugs using human behavioral pharmacology methods can be leveraged to help establish this link.

The primary goals of this brief perspective article are to (1) present a simple framework for integrating noninvasive brain stimulation with behavioral pharmacology techniques to better understand cognitive mechanisms and associated neural function and (2) provide some considerations for such an approach applied to the study of addiction. We use an ongoing study in our laboratory for illustration that combines delta-9-tetrahydrocannabinol (THC), the main psychoactive compound in cannabis, with functional magnetic resonance imaging (fMRI)-guided TMS to examine THC intoxication in individuals reporting problematic cannabis use. First, a simple framework for integrating TMS with human behavioral pharmacology is provided. Next, key details of the study are presented. We then discuss challenges, alternatives, and future directions. We envision that the information presented here will aid similar future approaches to advance the understanding and treatment of addiction.

### 1.1. Framework for integrating TMS with clinical pharmacology

**Figure 1:**
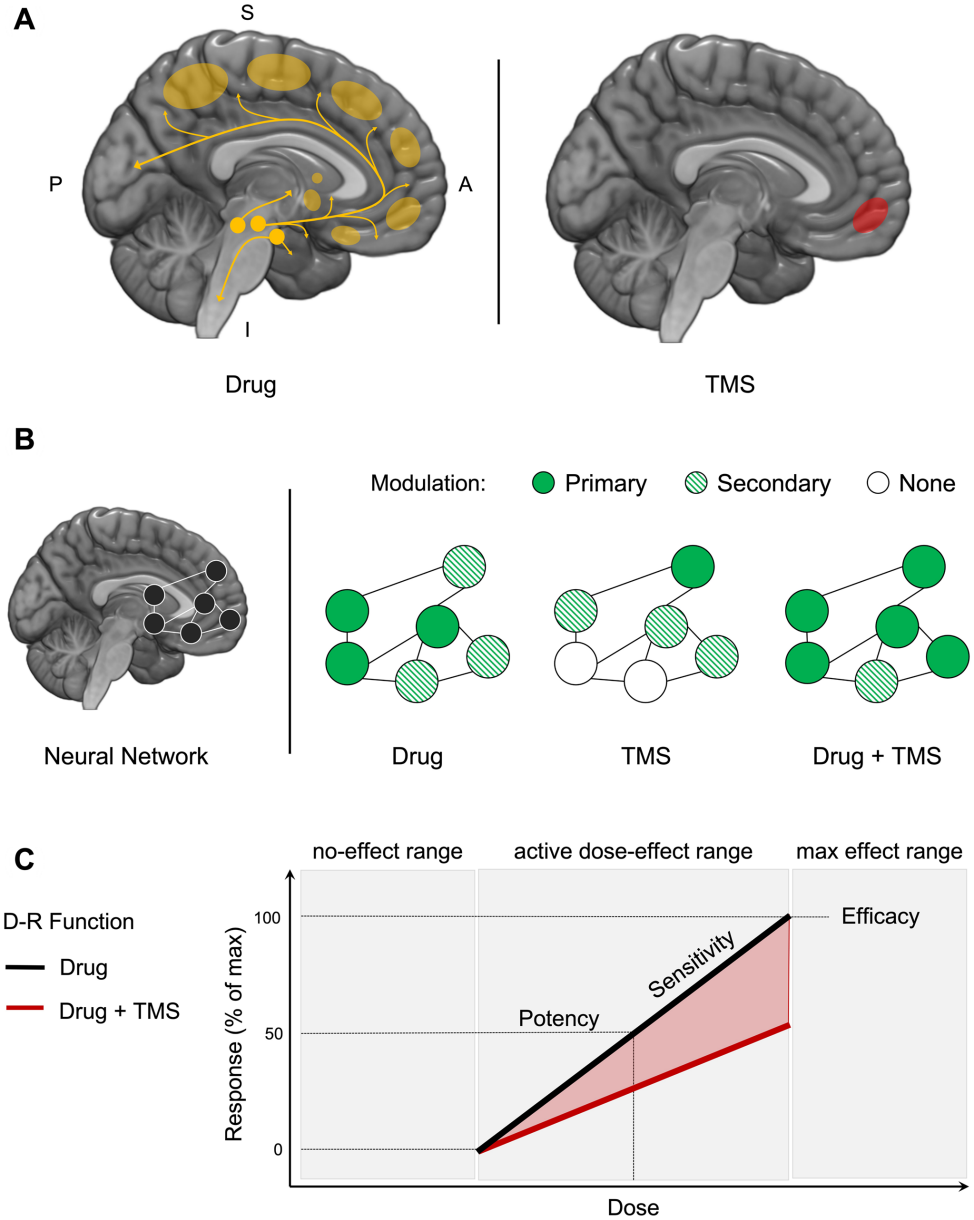
Framework for integrating noninvasive brain stimulation with behavioral pharmacology. **(A)** A drug yields its initial effect on molecular targets according to the wide spanning topography of its neurotransmitter system (left). Drugs of abuse are known to modulate function in dopaminergic neurons originating in the brainstem and projecting to striatal and neocortical brain regions. Whereas dopaminergic modulation in the ventral striatum has been largely linked to the reinforcing effects of abused drugs, modulation of the prefrontal cortex has been linked to deficits in executive functioning ability, including compromised learning, memory, and attention. TMS yields its initial effect on more molar targets with diverse molecular physiology (right). For example, stimulation of the medial prefrontal cortex (mPFC) can modulate the firing rates of primary neurons and interneurons containing a diverse array of excitatory and inhibitory neurotransmitters. Importantly, interactions in the effects of drugs and TMS can exist based on their ability to both modulate neurophysiology. **(B)** Across a neural network, function in specialized and interacting regions (nodes, hubs, modules) underlies the complex experiences and behaviors associated with problematic drug use (left). The combined administration of a psychotropic drug and noninvasive brain stimulation can interact within a region and/or across a neural network to have an impact on addiction-related outcomes (right). In a simple example, if decreased mPFC function is observed in response to a drug and associated with compromised learning ability, then pairing that drug with an excitatory stimulation protocol targeting the mPFC may recover learning ability. It is possible for interactions in modulation to occur in primary and secondary regions across a network. Studying such interactions holds promise for better understanding the cognitive mechanisms and associated brain function thought to underlie drug-related behavior. **(C)** In behavioral pharmacology, a drug dose–response (D-R) function characterizes the relationship between a drug’s dose and its biological impact on a behavioral response (black line). Potency, efficacy, and slope are the primary characteristics of the function. Potency describes the amount of a drug that must be taken to achieve a specific level of response, such as 50% of the maximum effect. Potency is measured by the lower concentration or dose necessary to produce a given response. Efficacy refers to a drug’s greatest effect. A drug that is more effective than another produces greater changes in a response. Sensitivity to changes in drug dose is reflected in the slope. A more sensitive system is represented by a steeper slope, whereas a less sensitive system is represented by a flatter slope. D-R functions can also be used to assess the safety of interventions, as well as to compare the efficacy of various interventions. The impact of TMS on a drug D-R function (red line) can reveal interactions in intervention modalities on cognitive outcomes and reveal knowledge about the involvement and plasticity of potential neural mechanisms targeted by TMS. In the current example, TMS delivered to a neural target decreases the sensitivity, potency, and efficacy of the drug effect on a behavioral response. P, posterior; A, anterior; S, superior; I, inferior.

A drug yields its initial effect on select molecular targets according to the wide spanning topography of its neurotransmitter system (Figure 1A, left). TMS yields its initial effect on more molar targets with diverse molecular physiology (Figure 1A, right). In network models of the brain, function in specialized and interacting regions underlies the behaviors that define SUDs (Watts and Strogatz, 1998; Sporns and Honey, 2006; Sporns et al., 2007; Telesford et al., 2011; Park and Friston, 2013). We theorize that the combination of a centrally acting drug and TMS will have a greater impact on network function to alter SUD outcomes (Figure 1B).

Prior pharmaco-TMS studies have typically established a TMS-behavior effect and then determined the impact of a drug on that effect, but here we describe how the effects of an abused drug on SUD-related behaviors can be established with human behavioral pharmacology methods and then used to investigate the role of a particular brain region on those behaviors using TMS. Human behavioral pharmacology methods have been used for over 50 years to reveal mechanisms of addiction and develop effective pharmacotherapies (Comer et al., 2010). In a typical behavioral pharmacology study, a dose–response (D-R) function is generated to characterize the relationship between a range of doses of an abused drug and a relevant behavioral response (Figure 1C). Pharmacologically selective intervention drugs can then be administered to assess shifts in drug D-R functions, which reveals information about the neurotransmitter systems involved in the behavior as well as the potential therapeutic use of the intervention drug (Brunton and Knollmann, 2023). This approach can be adapted for TMS, such that the influence of region-specific TMS on the efficacy, potency, and sensitivity of a drug effect on behavior can reveal the importance of that region in observed behavior (see Figure 1 legend for more detail). This design can also be used to inform the potential use of that TMS protocol as a therapeutic.

### 1.2. Participants

Cannabis use disorder is the focus of this study because rates of use and CUD diagnoses are on the rise (SAMHSA, 2021). Moreover, social acceptance of cannabis use and the availability of high potency commercial cannabinoid products (including novel analogs such as delta-8-THC) are at an all time high (Chandra et al., 2019; Daniller, 2019; Hartman, 2022; Johnston et al., 2022).

Participants are non-treatment seeking young adults aged 18–34 years old with problematic cannabis use, which is operationalized as (1) consuming cannabis on a daily/near daily basis (≥20 days/month) and (2) meeting hazardous use criteria according to the CUDIT-R (Adamson et al., 2010), and/or meeting CUD criteria according to the DSM-V (First et al., 2015). Exclusion criteria include medical screening outcomes that are abnormal or have the potential to interfere with study participation, including past or current serious physical disease, brain injury, or seizures. Past or current psychiatric disorder(s), including SUD other than cannabis or nicotine, and metal implants contraindicated for MRI.

### 1.3. Study outcomes

This study examines the role of the left dorsolateral prefrontal cortex (DLPFC) on THC intoxication defined by the impairing effects of THC on decision-making, working memory, and subjective outcomes. CUD is largely characterized by maladaptive decision-making, such as choosing to use cannabis at the exclusion of other behaviors and despite negative consequences (Zehra et al., 2018). Individuals with CUD frequently make decisions while intoxicated, which is problematic because cannabis/THC impairs decision-making performance (Liguori et al., 1998; Ramaekers et al., 2000; Lane et al., 2005b) and associated cognitive functions such as working memory and attention (Kelly et al., 1990; Greenwald and Stitzer, 2000; Ilan et al., 2004; Lane et al., 2005a). Moreover, impaired decision-making in cannabis users has been positively associated with cannabis use frequency and negative consequences of use (Gonzalez et al., 2012). Decision-making is being assessed using a probabilistic reinforcement-learning (RL) choice task (Rutledge et al., 2009). In this task, two options signaled by distinct cues are available and choosing either could result in the delivery of monetary reward, but the probabilities of the options differ, and change unpredictably during the task. Working memory is assessed using the N-Back task, which measures performance under different working memory loads. Network function during this task has been linked to increased future cannabis (Cousijn et al., 2014).

A Visual Analogue Scale (VAS) subjective effects questionnaire is included because the positive subjective effects of drugs are a measure of their abuse potential [i.e., likelihood of maintaining sustained nonmedical use; (Griffiths et al., 2003)]. With respect to cannabis, prior research found that individuals who displayed a more positive initial subjective experience with cannabis had a shorter latency to subsequent use, greater lifetime use and were more likely to develop disordered use (Davidson and Schenk, 1994; Fergusson et al., 2003; Le Strat et al., 2009). Our version of the task includes positive (e.g., like drug), negative (e.g., nauseated) and cannabis/THC-specific (e.g., high) items (Wesley et al., 2018).

### 1.4. Intervention protocols

The synthetic version of THC, dronabinol, is administered orally under double-blind conditions. Dronabinol is FDA-approved to treat HIV/AIDS-induced anorexia and chemotherapy-induced nausea and vomiting, but it is being used here as a pharmacological probe to establish THC-behavior effects. The off-label use of FDA-approved medications is commonplace in behavioral pharmacology studies. Oral administration was chosen to help maintain participant and research staff blindness and to eliminate expectations that might accompany other routes of administration. Participants receive over-encapsulated commercial dronabinol; placebo capsules contain a behaviorally inert substance (e.g., corn starch). The active doses of THC (10 and 30 mg) were chosen based on previous oral THC administration studies (Lile et al., 2010a, b, 2011, 2012, 2013, 2015). For comparison, the starting therapeutic dose is 2.5–5 mg, administered 4–6 times per day, which can be increased to 10-20 mg per dose. Plasma concentrations for oral THC peak between 2 and 4 h (Hollister et al., 1981). The half-life of THC is 19-36 h, but the duration of the behavioral effects is roughly 4–6 h (e.g., Lemberger et al., 1972 Hollister et al., 1981).

TMS is administered with the MagVenture Cool-B65 active/sham coil under double-blind conditions. The active protocol is intermittent theta burst stimulation (iTBS) applied to the left dorsolateral prefrontal cortex (DLPFC). The iTBS600 protocol is considered “excitatory” based on its ability to facilitate motor evoked potentials in the motor cortex (Huang et al., 2005; Wischnewski and Schutter, 2015). It consists of 20 trains of 3 pulses delivered at 50 Hz repeating at 200 ms intervals with 2 s on (30 pulses/train) and 8 s off over 190 s (Huang et al., 2005; Wischnewski and Schutter, 2015). Ten initial trains are administered that proportionally ramp up to the desired stimulation intensity. iTBS is delivered at 80% resting motor threshold (RMT) and expected to modulate function for approximately 20–60 m based on previous motor effects (Huang et al., 2005; Wischnewski and Schutter, 2015). Sham stimulation involves positioning the coil over the stimulation target with the active side facing outward. For both sham/active conditions, electrodes are placed approximately 4–5 cm apart on the scalp on either side of the stimulation trajectory. Electrodes pass subcutaneous currents in synchronization with the stimulation protocol to generate skin and auditory sensations that further facilitate blinding.

The left DLPFC was chosen as the TMS target because of existing data implicating this region in the cognitive impairing and subjective effects of cannabis/THC. Previous fMRI studies, including our own, have demonstrated that the left DLPFC is involved in decision-making, working memory, and attention processes (Wesley et al., 2011, 2014; Wesley and Bickel, 2014). Fronto-striatal circuits that involve the DLPFC have also been implicated in reinforcement-based computational models of learning and memory (Lipton et al., 2019; Volkow et al., 2019; Averbeck and O'Doherty, 2022; Liebenow et al., 2022). Consistent with a role in CUD, left DLPFC function predicted cannabis versus money choice (Bedi et al., 2015). Combining positron emission tomography or fMRI with left DLPFC TMS has demonstrated that stimulating this region causes molecular and functional changes, respectively, in executive control and striatal brain regions (Strafella et al., 2001; Pogarell et al., 2007; Cho and Strafella, 2009; Hanlon et al., 2013; Gorelick et al., 2014; Caparelli et al., 2022). The ability of TMS to modulate glutamate and dopamine function in mesocorticolimbic circuits is consistent with its use as a tool to link brain activity with abuse-related behavior and as a potential treatment for drug use disorder, including CUD (Gorelick et al., 2014; Hanlon et al., 2018; Steele, 2020b; Kearney-Ramos and Haney, 2021).

### 1.5. Experimental procedures and data analysis

A detailed account of all procedures is beyond the scope of this communication, so focus is given to those most relevant for TMS and drug combination studies enrolling individuals reporting nonmedical drug use. The study proper consists of 7 outpatient laboratory visits over approximately 3–5 weeks: 1 training/neuroimaging session and 6 sessions in which THC and iTBS are co-administered. Daily check-in and -out procedures follow those detailed elsewhere (Wesley et al., 2018). Briefly, field sobriety tests are conducted, and expired-breath samples are collected to detect recent alcohol use. Urine is tested for recent use of abused drugs with qualitative, commercially available kits. Participants must agree to abstain from nonmedical use of drugs other than cannabis for the duration of the study. Participants must also agree to abstain from using cannabis and alcohol for 12 h and ingesting solid food and caffeine for 4 h prior to each visit. They are provided a standard, fat- and caffeine-free snack during study visits.

**Figure 2:**
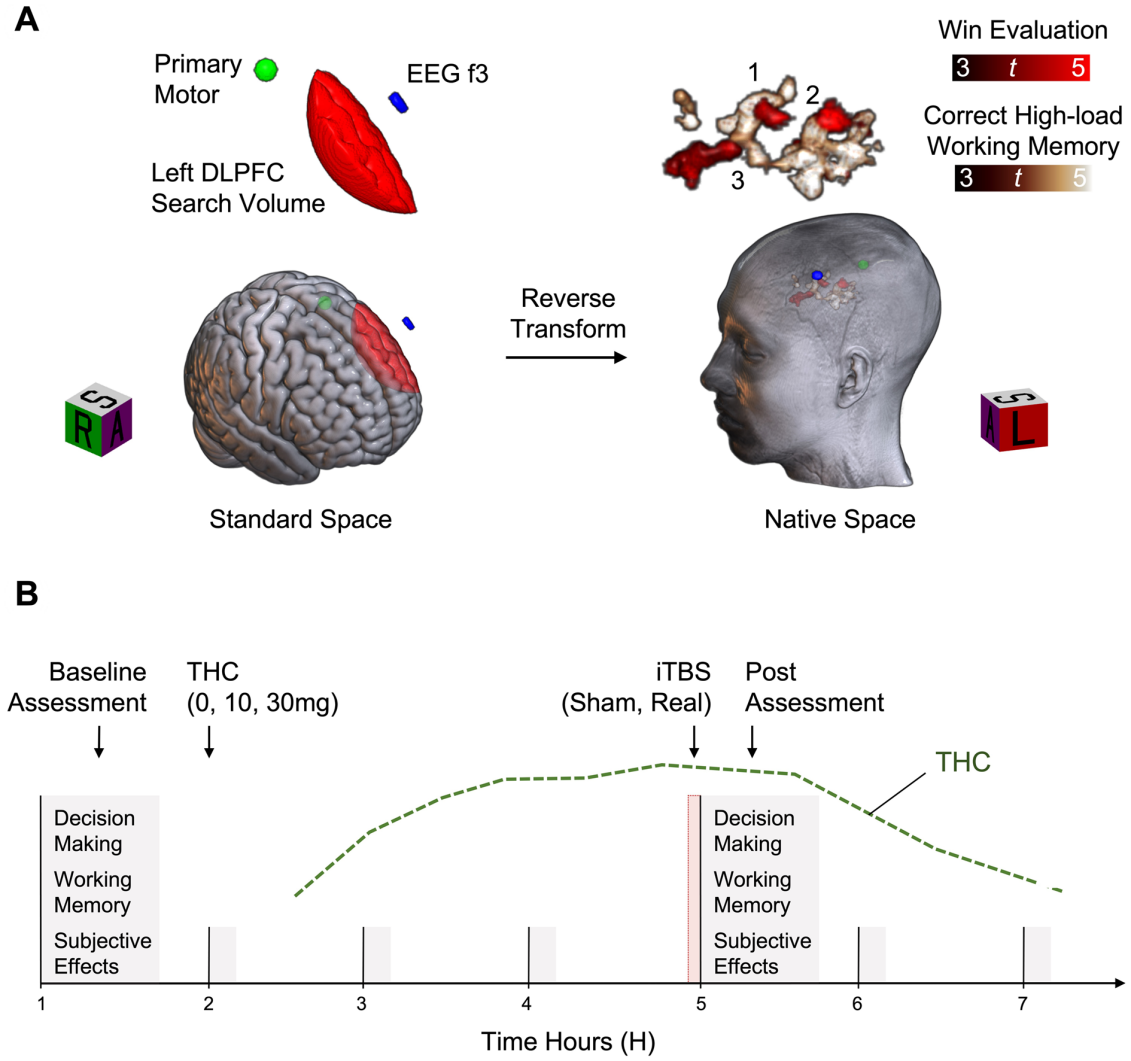
Example study design. **(A)** Functional Magnetic Resonance Imaging (fMRI) data are acquired during completion of decision-making and working memory tasks. For each participant, fMRI data are preprocessed and analyzed in standard space with fixed effects general linear models. Function associated with win evaluation on the decision-making task (Win Evaluation > Control Evaluation) and correct high-load working memory on the working memory task (High-Load > Low-Load) are isolated within a left dorsolateral prefrontal cortex (Left DLPFC) search volume. Results are reverse transformed into a participant’s native brain space, along with standard space locations of the EEG f3 scalp spot and the primary motor cortex location. Of the potential stimulation targets identified (3 shown), the target closest to the skull is selected for modulation. **(B)** Across six separate experimental sessions, all combinations of THC (0, 10, and 30 mg) and iTBS (sham, active) are tested. First, a baseline task assessment is completed, followed by capsule administration. iTBS is administered approximately 3 h after capsule administration, corresponding to the estimated peak of THC effects (green line). This is immediately followed by reassessment with the task battery.

The first visit lasts approximately 3–4 h and establishes the target for individualized TMS delivery (Figure 2A). Participants are trained on the decision-making and working memory tasks before performing them in the MRI scanner. For each participant, brain activity during each task is preprocessed and analyzed in standard brain space with fixed effects general linear models (Wesley et al., 2014, 2016, 2017). A left DLPFC explicit mask is used to isolate function associated with evaluating wins on the decision-making task and correct high-load performance on the working memory task (relative to task-specific control events). Results are reverse transformed into a participant’s native brain space, along with standard space locations of the EEG f3 scalp spot and the primary motor cortex location. The latter is used to initiate a grid search for calculating RMT. The stimulation target is the left DLPFC location of overlap closest to the skull that represents “good” task performance. Immediately after scanning, data are analyzed, RMT is calculated, and one train of active iTBS is administered to familiarize participants with the procedure. Neurotargeting is performed with commercially available equipment (Brainsight; Rogue Research Inc., Montreal, Quebec, Canada).

Six experimental sessions are conducted to test all possible combinations of THC (0, 10, and 30 mg) and iTBS (sham and active) (Figure 2B). These sessions last approximately 7 h each and are separated by a minimum of 2 days. Based on our previous THC administration studies (Lile et al., 2015; Wesley et al., 2018) and protocols from previous iTBS studies (Chung et al., 2018), 2 days was deemed sufficient to prevent the accumulation of carryover effects. Of note, while the minimum time between sessions was 2 days, most sessions in the ongoing study are separated by one to 2 weeks further mitigating the potential for carryover effects. Combinations are randomized except that 30 mg and active iTBS is not administered prior to 30 mg and sham iTBS, for safety. First, a baseline task assessment is completed, followed by capsule administration. The stimulation protocol is administered approximately 3 h after capsule administration, corresponding to the estimated peak effects of THC, followed by completion of the task battery. Vital signs are monitored every hour throughout experimental sessions. Prior to discharge, participants are assessed for residual drug effects and cautioned about potential impairing effects on subsequent activities.

Primary analyses focus on elucidating the involvement of the left DLPFC in the acute cognitive impairing and subjective effects of THC. To this end, the ability of active iTBS to impact the dose-dependent effects of THC on targeted outcomes is determined. Daily baseline assessments are used to calculate change scores for each outcome. Then, a THC (0, 10, and 30 mg) D-R curve for each iTBS condition (sham and active) is generated for each outcome. We hypothesize that if left DLPFC function is involved in the acute impairing and/or subjective effects of THC, then activation of this region by iTBS will reverse the effects of THC, as indicated by a shift in the THC D-R curve for targeted outcomes.

## 2. Discussion

A significant challenge in conducting research that involves TMS and the administration of abused drugs to human subjects is the expertise required. In addition to foundational training in experimental psychology, pharmacology, physiology, anatomy, neuroscience, and statistics, these studies require additional training in, and adherence to, Human Subjects Protections, Responsible Conduct of Research and Good Clinical Practice standards, as well as certification in TMS delivery. The neuroimaging and neurobehavioral modeling described here requires additional training and technical skills. Thoughtful design choices must integrate knowledge across a range of medical, scientific, and technical disciplines. Therefore, multidisciplinary teams working in highly collaborative and effective research and training environments are best suited for this work.

The study described here tests two active THC doses and a single active iTBS dose, and the inclusion of appropriate control conditions (placebo THC and sham TMS) doubles the number of experimental conditions and study sessions. Ideally, several active doses of each intervention modality would be administered, alone and in combination, to better capture the relationship between TMS and THC on abuse-related behaviors. However, increasing the number of conditions would extend study enrollment and likely increase study dropout. A between-subjects design could be utilized, but would be less rigorous, require more participants, and complicate interpretation of individual differences thought to be critical in SUD (George and Koob, 2017). Exciting new preclinical/clinical research is focused on establishing TMS D-R effects in neocortical targets using different stimulation modalities/protocols and neuroimaging techniques (INNN, 2022), which will guide future study designs by narrowing the parameter space for testable protocols to pair with drug administration. Furthermore, the integration of TMS with valid preclinical behavioral pharmacology models would inform future clinical research.

The findings from the current study may be less generalizable to the treatment of substance use disorders in clinical settings, given that participants are non-treatment seeking and that high rates of psychiatric comorbidities exist in substance-using populations. A related design consideration is whether to use acute administration of TMS or a clinical treatment protocol (i.e., daily/weekly and/or accelerated TMS) to examine the role of targeted regions in SUD (Ekhtiari et al., 2019; Steele et al., 2019). Using clinical treatment protocols would help to compare results from laboratory studies and clinical efficacy trials but raises further concerns about study retention due to the additional time required. Further, studies enrolling treatment-seeking individuals could not include abused drug administration, though putative pharmacotherapies could be combined with TMS in those trials to determine their efficacy to treat SUD, which represents another valuable TMS and drug administration approach.

This example study includes a limited number of behavioral outcomes, but there are several other outcomes relevant to SUD and results might differ in individuals seeking treatment. For example, craving is thought to be an important driver of continued drug use and clinical studies have demonstrated the ability of various stimulation modalities/protocols targeting prefrontal regions to impact craving in non-treatment-seeking and treatment-seeking individuals (Hanlon et al., 2015; Hone-Blanchet et al., 2015; Zhao et al., 2020). Interactions between drugs and stimulation modalities/protocols on craving and other outcomes could be examined in future work.

Lastly, we selected iTBS to combine with THC in our ongoing study because of its ability to directly impact activity in a relatively small neocortical functional target, but other stimulation modalities/protocols could also be considered. For example, transcranial electric current simulation has been paired with neuroimaging has been shown to impact SUD outcomes (Yavari et al., 2016; Ekhtiari et al., 2022). Future studies combining non-invasive brain stimulation and pharmacologically selective drugs might also target functional connectivity related to SUD-relevant outcomes.

## Data availability statement

The original contributions presented in the study are included in the article/supplementary material, further inquiries can be directed to the corresponding author.

## Author contributions

MW conceived the framework for the described study. MW and JL designed the described study and contributed to writing this perspective article. All authors contributed to the article and approved the submitted version.

## Funding

This work is funded by a NIDA career development and training award titled, “Neural Mechanisms of Cannabinoid-Impaired Decision-Making in Emerging Adults” (K01DA043652).

## Conflict of interest

The authors declare that the research was conducted in the absence of any commercial or financial relationships that could be construed as a potential conflict of interest.

## Publisher’s note

All claims expressed in this article are solely those of the authors and do not necessarily represent those of their affiliated organizations, or those of the publisher, the editors and the reviewers. Any product that may be evaluated in this article, or claim that may be made by its manufacturer, is not guaranteed or endorsed by the publisher.

## References

[ref1] Adamson S. J. Kay-Lambkin F. J. Baker A. L. Lewin T. J. Thornton L. Kelly B. J.. (2010). An improved brief measure of cannabis misuse: the Cannabis use disorders identification test-revised (CUDIT-R). Drug Alcohol Depend. 110, 137–143. doi: 10.1016/j.drugalcdep.2010.02.017, PMID: 20347232

[ref2] Averbeck B. O'Doherty J. P. (2022). Reinforcement-learning in fronto-striatal circuits. Neuropsychopharmacology 47, 147–162. doi: 10.1038/s41386-021-01108-0, PMID: 34354249 PMC8616931

[ref3] Bedi G. Lindquist M. A. Haney M. (2015). An fMRI-based neural signature of decisions to smoke Cannabis. Neuropsychopharmacology 40, 2657–2665. doi: 10.1038/npp.2015.135, PMID: 25962875 PMC4864661

[ref4] BrainsWay (2020). BrainsWay receives FDA clearance for smoking addiction in adults. [Press Release] [Online]. Available at: https://www.globenewswire.com/news-release/2020/08/24/2082476/0/en/BrainsWay-Receives-FDA-Clearance-for-Smoking-Addiction-in-Adults.html

[ref5] Brunton L. L. Knollmann B. r. C. (2023). Goodman & Gilman's the pharmacological basis of therapeutics. New York: McGraw Hill.

[ref6] Caparelli E. C. Schleyer B. Zhai T. Gu H. Abulseoud O. A. Yang Y. (2022). High-frequency transcranial magnetic stimulation combined with functional magnetic resonance imaging reveals distinct activation patterns associated with different dorsolateral prefrontal cortex stimulation sites. Neuromodulation 25, 633–643. doi: 10.1016/j.neurom.2022.03.002, PMID: 35418339

[ref7] Ceceli A. O. Bradberry C. W. Goldstein R. Z. (2022). The neurobiology of drug addiction: cross-species insights into the dysfunction and recovery of the prefrontal cortex. Neuropsychopharmacology 47, 276–291. doi: 10.1038/s41386-021-01153-9, PMID: 34408275 PMC8617203

[ref8] Chandra S. Radwan M. M. Majumdar C. G. Church J. C. Freeman T. P. ElSohly M. A. (2019). New trends in cannabis potency in USA and Europe during the last decade (2008–2017). Eur. Arch. Psychiatry Clin. Neurosci. 269, 5–15. doi: 10.1007/s00406-019-00983-5, PMID: 30671616

[ref9] Chipchase L. Schabrun S. Cohen L. Hodges P. Ridding M. Rothwell J.. (2012). A checklist for assessing the methodological quality of studies using transcranial magnetic stimulation to study the motor system: an international consensus study. Clin. Neurophysiol. 123, 1698–1704. doi: 10.1016/j.clinph.2012.05.003, PMID: 22647458 PMC4884647

[ref10] Cho S. S. Strafella A. P. (2009). rTMS of the left dorsolateral prefrontal cortex modulates dopamine release in the ipsilateral anterior cingulate cortex and orbitofrontal cortex. PLoS One 4:e6725. doi: 10.1371/journal.pone.0006725, PMID: 19696930 PMC2725302

[ref11] Chung S. W. Rogasch N. C. Hoy K. E. Sullivan C. M. Cash R. F. H. Fitzgerald P. B. (2018). Impact of different intensities of intermittent theta burst stimulation on the cortical properties during TMS-EEG and working memory performance. Hum. Brain Mapp. 39, 783–802. doi: 10.1002/hbm.23882, PMID: 29124791 PMC6866298

[ref12] Cipriani A. Furukawa T. A. Salanti G. Chaimani A. Atkinson L. Z. Ogawa Y.. (2018). Comparative efficacy and acceptability of 21 antidepressant drugs for the acute treatment of adults with major depressive disorder: a systematic review and network meta-analysis. Lancet 391, 1357–1366. doi: 10.1016/S0140-6736(17)32802-7, PMID: 29477251 PMC5889788

[ref13] Coles A. S. Kozak K. George T. P. (2018). A review of brain stimulation methods to treat substance use disorders. Am. J. Addict. 27, 71–91. doi: 10.1111/ajad.12674, PMID: 29457674 PMC6034717

[ref14] Comer S. D. Bickel W. K. Yi R. de Wit H. Higgins S. T. Wenger G. R.. (2010). Human behavioral pharmacology, past, present, and future: symposium presented at the 50th annual meeting of the behavioral pharmacology society. Behav. Pharmacol. 21, 251–277. doi: 10.1097/FBP.0b013e32833bb9f8, PMID: 20664330 PMC2913311

[ref15] Cousijn J. Wiers R. W. Ridderinkhof K. R. van den Brink W. Veltman D. J. Goudriaan A. E. (2014). Effect of baseline cannabis use and working-memory network function on changes in cannabis use in heavy cannabis users: a prospective fMRI study. Hum. Brain Mapp. 35, 2470–2482. doi: 10.1002/hbm.22342, PMID: 24038570 PMC6869744

[ref16] Daniller A. (2019). Two-thirds of Americans support marijuana legalization. Pew Research Center. Available at: https://www.pewresearch.org/fact-tank/2019/11/14/americans-support-marijuana-legalization/

[ref17] Davidson E. S. Schenk S. (1994). Variability in subjective responses to marijuana: initial experiences of college students. Addict. Behav. 19, 531–538. doi: 10.1016/0306-4603(94)90008-6, PMID: 7832011

[ref18] de Moraes Costa G. Zanatta F. B. Ziegelmann P. K. Soares Barros A. J. Mello C. F. (2020). Pharmacological treatments for adults with post-traumatic stress disorder: a network meta-analysis of comparative efficacy and acceptability. J. Psychiatr. Res. 130, 412–420. doi: 10.1016/j.jpsychires.2020.07.046, PMID: 32891916

[ref19] Dunlop K. Hanlon C. A. Downar J. (2017). Noninvasive brain stimulation treatments for addiction and major depression. Ann. N. Y. Acad. Sci. 1394, 31–54. doi: 10.1111/nyas.12985, PMID: 26849183 PMC5434820

[ref20] Ekhtiari H. Soleimani G. Kuplicki R. Yeh H. W. Cha Y. H. Paulus M. (2022). Transcranial direct current stimulation to modulate fMRI drug cue reactivity in methamphetamine users: a randomized clinical trial. Hum. Brain Mapp. 43, 5340–5357. doi: 10.1002/hbm.26007, PMID: 35915567 PMC9812244

[ref21] Ekhtiari H. Tavakoli H. Addolorato G. Baeken C. Bonci A. Campanella S.. (2019). Transcranial electrical and magnetic stimulation (tES and TMS) for addiction medicine: a consensus paper on the present state of the science and the road ahead. Neurosci. Biobehav. Rev. 104, 118–140. doi: 10.1016/j.neubiorev.2019.06.007, PMID: 31271802 PMC7293143

[ref22] Feil J. Zangen A. (2010). Brain stimulation in the study and treatment of addiction. Neurosci. Biobehav. Rev. 34, 559–574. doi: 10.1016/j.neubiorev.2009.11.006, PMID: 19914283

[ref23] Fergusson D. M. Horwood L. J. Lynskey M. T. Madden P. A. (2003). Early reactions to cannabis predict later dependence. Arch. Gen. Psychiatry 60, 1033–1039. doi: 10.1001/archpsyc.60.10.1033, PMID: 14557149

[ref24] First M. B. Williams J. B. W. Karg R. S. Spitzer R. L. (2015). "Structured clinical interview for DSM-5—Research version (SCID-5 for DSM-5, research version; SCID-5-RV)", American Psychiatric Association Arlington, VA.

[ref25] George O. Koob G. F. (2017). Individual differences in the neuropsychopathology of addiction. Dialogues Clin. Neurosci. 19, 217–229. doi: 10.31887/DCNS.2017.19.3/gkoob, PMID: 29302219 PMC5741105

[ref26] Gonzalez R. Schuster R. M. Mermelstein R. J. Vassileva J. Martin E. M. Diviak K. R. (2012). Performance of young adult cannabis users on neurocognitive measures of impulsive behavior and their relationship to symptoms of cannabis use disorders. J. Clin. Exp. Neuropsychol. 34, 962–976. doi: 10.1080/13803395.2012.703642, PMID: 22882144 PMC3488122

[ref27] Gorelick D. A. Zangen A. George M. S. (2014). Transcranial magnetic stimulation in the treatment of substance addiction. Ann. N. Y. Acad. Sci. 1327, 79–93. doi: 10.1111/nyas.12479, PMID: 25069523 PMC4206564

[ref28] Greenwald M. K. Stitzer M. L. (2000). Antinociceptive, subjective and behavioral effects of smoked marijuana in humans. Drug Alcohol Depend. 59, 261–275. doi: 10.1016/S0376-8716(99)00128-3, PMID: 10812286

[ref29] Griffiths R. R. Bigelow G. E. Ator N. A. (2003). Principles of initial experimental drug abuse liability assessment in humans. Drug Alcohol Depend. 70, S41–S54. doi: 10.1016/s0376-8716(03)00098-x, PMID: 12759196

[ref30] Hanlon C. A. Canterberry M. Taylor J. J. DeVries W. Li X. Brown T. R.. (2013). Probing the frontostriatal loops involved in executive and limbic processing via interleaved TMS and functional MRI at two prefrontal locations: a pilot study. PLoS One 8:e67917. doi: 10.1371/journal.pone.0067917, PMID: 23874466 PMC3706588

[ref31] Hanlon C. A. Dowdle L. T. Austelle C. W. DeVries W. Mithoefer O. Badran B. W.. (2015). What goes up, can come down: novel brain stimulation paradigms may attenuate craving and craving-related neural circuitry in substance dependent individuals. Brain Res. 1628, 199–209. doi: 10.1016/j.brainres.2015.02.053, PMID: 25770818 PMC4899830

[ref32] Hanlon C. A. Dowdle L. T. Henderson J. S. (2018). Modulating neural circuits with transcranial magnetic stimulation: implications for addiction treatment development. Pharmacol. Rev. 70, 661–683. doi: 10.1124/pr.116.013649, PMID: 29945899 PMC6020107

[ref33] Harris A. Reece J. (2021). Transcranial magnetic stimulation as a treatment for posttraumatic stress disorder: a meta-analysis. J. Affect. Disord. 289, 55–65. doi: 10.1016/j.jad.2021.04.003, PMID: 33940319

[ref34] Hartman M. (2022). Cannabis overview: national conference on state legislatures. Available at: https://www.ncsl.org/research/civil-and-criminal-justice/marijuana-overview.aspx#:~:text=Twenty%2Dseven%20states%20and%20the,no%20possibility%20of%20jail%20time

[ref1007] Hollister L. E. Gillespie H. K. Ohlsson A. Lindgren J. E. Wahlen A. Agurell S. (1981). Do plasma concentrations of delta 9-tetrahydrocannabinol reflect the degree of intoxication?. J. Clin. Pharmacol. 21, 171S–177S. doi: 10.1002/j.1552-4604.1981 6271822

[ref35] Hone-Blanchet A. Ciraulo D. A. Pascual-Leone A. Fecteau S. (2015). Noninvasive brain stimulation to suppress craving in substance use disorders: review of human evidence and methodological considerations for future work. Neurosci. Biobehav. Rev. 59, 184–200. doi: 10.1016/j.neubiorev.2015.10.001, PMID: 26449761 PMC5365234

[ref36] Huang Y. Z. Edwards M. J. Rounis E. Bhatia K. P. Rothwell J. C. (2005). Theta burst stimulation of the human motor cortex. Neuron 45, 201–206. doi: 10.1016/j.neuron.2004.12.033 15664172

[ref37] Ilan A. B. Smith M. E. Gevins A. (2004). Effects of marijuana on neurophysiological signals of working and episodic memory. Psychopharmacology 176, 214–222. doi: 10.1007/s00213-004-1868-9, PMID: 15502936 PMC1463999

[ref38] INNN (2022). International network on neuroimaging neuromodulation (INNN). Dose-response in non-invasive brain stimulation (online). Available at: https://www.youtube.com/@INNN_Network/playlists (Accessed January 18, 2023).

[ref39] Johnston L. D. Miech R. A. O’Malley P. M. Bachman J. G. Schulenberg J. E. Patrick M. E. (2022)." Monitoring the future national survey results on drug use 1975–2021: Overview, key findings on adolescent drug use."Ann Arbor: Institute for Social Research, University of Michigan.

[ref40] Kearney-Ramos T. Haney M. (2021). Repetitive transcranial magnetic stimulation as a potential treatment approach for cannabis use disorder. Prog. Neuro-Psychopharmacol. Biol. Psychiatry 109:110290. doi: 10.1016/j.pnpbp.2021.110290, PMID: 33677045 PMC9165758

[ref41] Kelly T. H. Foltin R. W. Rose A. J. Fischman M. W. Brady J. V. (1990). Smoked marijuana effects on tobacco cigarette smoking behavior. J. Pharmacol. Exp. Ther. 252, 934–944. PMID: 2319477

[ref42] Koob G. F. Volkow N. D. (2016). Neurobiology of addiction: a neurocircuitry analysis. Lancet Psychiatry 3, 760–773. doi: 10.1016/S2215-0366(16)00104-8, PMID: 27475769 PMC6135092

[ref43] Korchounov A. Ziemann U. (2011). Neuromodulatory neurotransmitters influence LTP-like plasticity in human cortex: a pharmaco-TMS study. Neuropsychopharmacology 36, 1894–1902. doi: 10.1038/npp.2011.75, PMID: 21544070 PMC3154108

[ref44] Lane S. D. Cherek D. R. Lieving L. M. Tcheremissine O. V. (2005a). Marijuana effects on human forgetting functions. J. Exp. Anal. Behav. 83, 67–83. doi: 10.1901/jeab.2005.22-04, PMID: 15762381 PMC1193701

[ref45] Lane S. D. Cherek D. R. Tcheremissine O. V. Lieving L. M. Pietras C. J. (2005b). Acute marijuana effects on human risk taking. Neuropsychopharmacology 30, 800–809. doi: 10.1038/sj.npp.1300620 15775958

[ref1006] Lemberger L. Weiss J. L. Watanabe A. M. Galanter I. M. Wyatt R. J. Cardon P .V. (1972). Delta-9-tetrahydrocannabinol. Temporal correlation of the psychologic effects and blood levels after various routes of administration. N. Engl. J. Med. 286, 685–688. doi: 10.1056/NEJM197203302861303 5061055

[ref46] Le Strat Y. Ramoz N. Horwood J. Falissard B. Hassler C. Romo L.. (2009). First positive reactions to cannabis constitute a priority risk factor for cannabis dependence. Addiction 104, 1710–1717. doi: 10.1111/j.1360-0443.2009.02680.x, PMID: 19663900

[ref47] Liebenow B. Jones R. DiMarco E. Trattner J. D. Humphries J. Sands L. P.. (2022). Computational reinforcement learning, reward (and punishment), and dopamine in psychiatric disorders. Front. Psych. 13:886297. doi: 10.3389/fpsyt.2022.886297, PMID: 36339844 PMC9630918

[ref1003] Lile J. A. Babalonis S. Emurian C. Martin C. A. Wermeling D. P. Kelly T. H. (2011). Comparison of the behavioral and cardiovascular effects of intranasal and oral d-amphetamine in healthy human subjects. J. Clin. Pharmacol. 51, 888–898. doi: 10.1177/0091270010375956 20671295 PMC3684273

[ref1005] Lile J. A. Kelly T. H. Charnigo R. J. Stinchcomb A. L. Hays L. R. (2013). Pharmacokinetic and pharmacodynamic profile of supratherapeutic oral doses of Delta(9) -THC in cannabis users. J. Clin. Pharmacol. 53, 680–690. doi: 10.1002/jcph.90 23754596 PMC3691290

[ref1001] Lile J. A. Kelly T. H. Hays L. R. (2010a). The reinforcing, self-reported performance and physiological effects of Delta9-tetrahydrocannabinol, triazolam, hydromorphone, and methylphenidate in cannabis users. Behav. Pharmacol. 21, 29–38. doi: 10.1097/FBP.0b013e32833470d7 19949319 PMC2903043

[ref1002] Lile J. A. Kelly T. H. Hays L. R. (2010b). Substitution profile of the cannabinoid agonist nabilone in human subjects discriminating delta9-tetrahydrocannabinol. Clin. Neuropharmacol. 33, 235–242. doi: 10.1097/WNF.0b013e3181e77428 20838217

[ref1004] Lile J. A. Kelly T. H. Hays L. R. (2012). Separate and combined effects of the GABA reuptake inhibitor tiagabine and Delta9-THC in humans discriminating Delta9-THC. Drug. Alcohol. Depend. 122, 61–69. doi: 10.1016/j.drugalcdep.2011.09.010 21975195 PMC3307819

[ref48] Liguori A. Gatto C. P. Robinson J. H. (1998). Effects of marijuana on equilibrium, psychomotor performance, and simulated driving. Behav. Pharmacol. 9, 599–609. doi: 10.1097/00008877-199811000-00015, PMID: 9862085

[ref49] Lile J. A. Wesley M. J. Kelly T. H. Hays L. R. (2015). Separate and combined effects of gabapentin and [INCREMENT]9-tetrahydrocananbinol in humans discriminating [INCREMENT]9-tetrahydrocananbinol. Behav. Pharmacol. 27, 215–224. doi: 10.1097/FBP.0000000000000187, PMID: 26313650 PMC4769128

[ref50] Lipton D. M. Gonzales B. J. Citri A. (2019). Dorsal striatal circuits for habits, compulsions and addictions. Front. Syst. Neurosci. 13:28. doi: 10.3389/fnsys.2019.00028, PMID: 31379523 PMC6657020

[ref51] Nagano T. Katsurada M. Yasuda Y. Kobayashi K. Nishimura Y. (2019). Current pharmacologic treatments for smoking cessation and new agents undergoing clinical trials. Ther. Adv. Respir. Dis. 13:1753466619875925. doi: 10.1177/1753466619875925, PMID: 31533544 PMC6755639

[ref52] Nitsche M. A. Muller-Dahlhaus F. Paulus W. Ziemann U. (2012). The pharmacology of neuroplasticity induced by non-invasive brain stimulation: building models for the clinical use of CNS active drugs. J. Physiol. 590, 4641–4662. doi: 10.1113/jphysiol.2012.232975, PMID: 22869014 PMC3487028

[ref53] Park H. J. Friston K. (2013). Structural and functional brain networks: from connections to cognition. Science 342:1238411. doi: 10.1126/science.1238411 24179229

[ref54] Perera T. George M. S. Grammer G. Janicak P. G. Pascual-Leone A. Wirecki T. S. (2016). The clinical TMS Society consensus review and treatment recommendations for TMS therapy for major depressive disorder. Brain Stimul. 9, 336–346. doi: 10.1016/j.brs.2016.03.010, PMID: 27090022 PMC5612370

[ref55] Pittenger C. Bloch M. H. (2014). Pharmacological treatment of obsessive-compulsive disorder. Psychiatr. Clin. North Am. 37, 375–391. doi: 10.1016/j.psc.2014.05.006, PMID: 25150568 PMC4143776

[ref56] Pogarell O. Koch W. Popperl G. Tatsch K. Jakob F. Mulert C.. (2007). Acute prefrontal rTMS increases striatal dopamine to a similar degree as D-amphetamine. Psychiatry Res. 156, 251–255. doi: 10.1016/j.pscychresns.2007.05.002, PMID: 17993266

[ref57] Ramaekers J. G. Robbe H. W. O'Hanlon J. F. (2000). Marijuana, alcohol and actual driving performance. Hum. Psychopharmacol. 15, 551–558. doi: 10.1002/1099-1077(200010)15:7<551::AID-HUP236>3.0.CO;2-P, PMID: 12404625

[ref58] Rapinesi C. Kotzalidis G. D. Ferracuti S. Sani G. Girardi P. Del Casale A. (2019). Brain stimulation in obsessive-compulsive disorder (OCD): a systematic review. Curr. Neuropharmacol. 17, 787–807. doi: 10.2174/1570159X17666190409142555, PMID: 30963971 PMC7059162

[ref59] Rutledge R. B. Lazzaro S. C. Lau B. Myers C. E. Gluck M. A. Glimcher P. W. (2009). Dopaminergic drugs modulate learning rates and perseveration in Parkinson's patients in a dynamic foraging task. J. Neurosci. 29, 15104–15114. doi: 10.1523/JNEUROSCI.3524-09.2009, PMID: 19955362 PMC3376711

[ref60] SAMHSA (2021). "SAMHSA", in: key substance use and mental health indicators in the United States: Results from the 2020 National Survey on drug use and health (HHS publication no. PEP21-07-01-003, NSDUH series H-56). Rockville, MD: Center for Behavioral Health Statistics and Quality, Substance Abuse and Mental Health Services Administration.

[ref61] Spagnolo P. A. Goldman D. (2017). Neuromodulation interventions for addictive disorders: challenges, promise, and roadmap for future research. Brain 140, aww284–aww1203. doi: 10.1093/brain/aww284, PMID: 28082299 PMC6059187

[ref62] Sporns O. Honey C. J. (2006). Small worlds inside big brains. Proc. Natl. Acad. Sci. U. S. A. 103, 19219–19220. doi: 10.1073/pnas.0609523103, PMID: 17159140 PMC1748207

[ref63] Sporns O. Honey C. J. Kotter R. (2007). Identification and classification of hubs in brain networks. PLoS One 2:e1049. doi: 10.1371/journal.pone.0001049, PMID: 17940613 PMC2013941

[ref64] Steele V. R. (2020a). Transcranial magnetic stimulation and addiction: toward uncovering known unknowns. EBioMedicine 57:102839. doi: 10.1016/j.ebiom.2020.102839, PMID: 32629385 PMC7334787

[ref65] Steele V. R. (2020b). Transcranial magnetic stimulation as an interventional tool for addiction. Front. Neurosci. 14:592343. doi: 10.3389/fnins.2020.592343, PMID: 33192278 PMC7641952

[ref66] Steele V. R. Maxwell A. M. Ross T. J. Stein E. A. Salmeron B. J. (2019). Accelerated intermittent Theta-burst stimulation as a treatment for cocaine use disorder: a proof-of-concept study. Front. Neurosci. 13:1147. doi: 10.3389/fnins.2019.01147, PMID: 31736689 PMC6831547

[ref67] Strafella A. P. Paus T. Barrett J. Dagher A. (2001). Repetitive transcranial magnetic stimulation of the human prefrontal cortex induces dopamine release in the caudate nucleus. J. Neurosci. 21:RC157. doi: 10.1523/JNEUROSCI.21-15-j0003.2001, PMID: 11459878 PMC6762641

[ref68] Telesford Q. K. Joyce K. E. Hayasaka S. Burdette J. H. Laurienti P. J. (2011). The ubiquity of small-world networks. Brain Connect. 1, 367–375. doi: 10.1089/brain.2011.0038, PMID: 22432451 PMC3604768

[ref69] Uhl G. R. Koob G. F. Cable J. (2019). The neurobiology of addiction. Ann. N. Y. Acad. Sci. 1451, 5–28. doi: 10.1111/nyas.13989, PMID: 30644552 PMC6767400

[ref70] Volkow N. D. Michaelides M. Baler R. (2019). The neuroscience of drug reward and addiction. Physiol. Rev. 99, 2115–2140. doi: 10.1152/physrev.00014.2018, PMID: 31507244 PMC6890985

[ref71] Watts D. J. Strogatz S. H. (1998). Collective dynamics of 'small-world' networks. Nature 393, 440–442. doi: 10.1038/30918 9623998

[ref72] Wesley M. J. Bickel W. K. (2014). Remember the future II: Meta-analyses and functional overlap of working memory and delay discounting. Biol. Psychiatry 75, 435–448. doi: 10.1016/j.biopsych.2013.08.008, PMID: 24041504 PMC3943930

[ref73] Wesley M. J. Hanlon C. A. Porrino L. J. (2011). Poor decision-making by chronic marijuana users is associated with decreased functional responsiveness to negative consequences. Psychiatry Res. 191, 51–59. doi: 10.1016/j.pscychresns.2010.10.002, PMID: 21145211 PMC3125637

[ref74] Wesley M. J. Lile J. A. Fillmore M. T. Porrino L. J. (2017). Neurophysiological capacity in a working memory task differentiates dependent from nondependent heavy drinkers and controls. Drug Alcohol Depend. 175, 24–35. doi: 10.1016/j.drugalcdep.2017.01.029, PMID: 28376413 PMC5425311

[ref75] Wesley M. J. Lile J. A. Hanlon C. A. Porrino L. J. (2016). Abnormal medial prefrontal cortex activity in heavy cannabis users during conscious emotional evaluation. Psychopharmacology 233, 1035–1044. doi: 10.1007/s00213-015-4180-y, PMID: 26690589 PMC4761289

[ref76] Wesley M. J. Lohrenz T. Koffarnus M. N. McClure S. M. De La Garza R. 2nd Salas R.. (2014). Choosing money over drugs: the neural underpinnings of difficult choice in chronic cocaine users. J. Addict. 2014:189853. doi: 10.1155/2014/189853, PMID: 25197609 PMC4150492

[ref77] Wesley M. J. Westgate P. M. Stoops W. W. Kelly T. H. Hays L. R. Lile J. A. (2018). Influence of tiagabine maintenance on cannabis effects and related behaviors in daily cannabis users. Exp. Clin. Psychopharmacol. 26, 310–319. doi: 10.1037/pha0000180, PMID: 29863387 PMC5990026

[ref78] Wischnewski M. Schutter D. J. (2015). Efficacy and time course of Theta burst stimulation in healthy humans. Brain Stimul. 8, 685–692. doi: 10.1016/j.brs.2015.03.004, PMID: 26014214

[ref79] Yavari F. Shahbabaie A. Leite J. Carvalho S. Ekhtiari H. Fregni F. (2016). Noninvasive brain stimulation for addiction medicine: from monitoring to modulation. Prog. Brain Res. 224, 371–399. doi: 10.1016/bs.pbr.2015.08.007 26822367

[ref80] Zehra A. Burns J. Liu C. K. Manza P. Wiers C. E. Volkow N. D.. (2018). Cannabis addiction and the brain: a review. J. Neuroimmune Pharmacol. 13, 438–452. doi: 10.1007/s11481-018-9782-9, PMID: 29556883 PMC6223748

[ref81] Zhao D. Li Y. Liu T. Voon V. Yuan T. F. (2020). Twice-daily Theta burst stimulation of the dorsolateral prefrontal cortex reduces methamphetamine craving: a pilot study. Front. Neurosci. 14:208. doi: 10.3389/fnins.2020.00208, PMID: 32273837 PMC7113524

[ref82] Ziemann U. (2011). Transcranial magnetic stimulation at the interface with other techniques: a powerful tool for studying the human cortex. Neuroscientist 17, 368–381. doi: 10.1177/1073858410390225 21311054

[ref83] Ziemann U. (2013). Pharmaco-transcranial magnetic stimulation studies of motor excitability. Handb. Clin. Neurol. 116, 387–397. doi: 10.1016/B978-0-444-53497-2.00032-2, PMID: 24112911

